# Testing Associations of Plant Functional Diversity with Carbon and Nitrogen Storage along a Restoration Gradient of Sandy Grassland

**DOI:** 10.3389/fpls.2016.00189

**Published:** 2016-02-19

**Authors:** Xiaoan Zuo, Xin Zhou, Peng Lv, Xueyong Zhao, Jing Zhang, Shaokun Wang, Xiyuan Yue

**Affiliations:** ^1^Naiman Desertification Research Station, Cold and Arid Regions Environmental and Engineering Research Institute, Chinese Academy of SciencesLanzhou, China; ^2^Laboratory of Stress Ecophysiology and Biotechnology, Cold and Arid Regions Environmental and Engineering Research Institute, Chinese Academy of SciencesLanzhou, China

**Keywords:** functional traits, functional dispersion, mass ratio hypothesis, multi-trait index, niche complementarity, vegetation restoration

## Abstract

The trait-based approach shows that ecosystem function is strongly affected by plant functional diversity as reflected by the traits of the most abundant species (community-weighted mean, CWM) and functional dispersion (FDis). Effects of CWM and FDis individually support the biomass ratio hypothesis and the niche complementarity hypothesis. However, there is little empirical evidence on the relative roles of CWM traits and FDis in explaining the carbon (C) and nitrogen (N) storage in grassland ecosystems. We measured plant functional traits in the 34 most abundant species across 24 sites along a restoration gradient of sandy grassland (mobile dune, semi-fixed dune, fixed dune, and grassland) in Horqin Sand Land, northern China. Thereafter, we calculated the CWM traits, the functional divergence of each single trait (FDvar) and the trait dispersion of multiple traits (FDis). We also measured the C and N storage in plant, litter, root, and soil. Using a stepwise multiple regression analysis, we further assessed which of the functional diversity components best explained C and N storage in the sandy grassland restoration. We found consistent links between C or N storage and leaf traits related to plant resource use strategy. However, the CWM of plant height was retained as an important predictor of C and N storage in plant, litter, soil, and total ecosystem in the final multiple models. CWMs of specific leaf area and plant height best predicted soil C and N storage and total ecosystem N storage. FDis was one of good predictors of litter C and N storage as well as total ecosystem C storage. These results suggest that ecosystem C and N pools in the sandy grassland restoration are primarily associated with the traits of the most abundant species in communities, thereby supporting the biomass ratio hypothesis. The positive associations of FDis with C storage in litter and total ecosystem provide evidence to support the niche complementarity hypothesis. Both functional traits of dominant species and traits’ dispersion in plant communities could contribute to explaining total ecosystem C storage. Thus, single- and multi-trait indices of functional composition play a crucial role in predicting C storage in sandy grasslands.

## Introduction

Accelerating declines in species diversity of terrestrial ecosystem have greatly affected ecosystem function (Butchart et al., 2010; Selmants et al., 2013). Many components of species diversity are used to access the relationship between biodiversity and ecosystem function (Tilman, 1997; Díaz et al., 2007; Mace et al., 2012). Increasing species richness may increase the space for the functional differences among species that strongly affect ecosystem processes (Petchey et al., 2004; Casanoves et al., 2011). The trait-based functional diversity, broadly defined as the value, range, and relative abundance of plant functional traits in a given community, is widely used in exploring plant community assembly (Bhaskar et al., 2014; Spasojevic et al., 2014), ecosystem function (Suding et al., 2008; Mouillot et al., 2011) and biogeochemical cycling processes (Conti and Díaz, 2013; Silva et al., 2013). Therefore, understanding associations of plant functional diversity with ecosystem function and properties can provide important insights into the underlying mechanisms of biodiversity effects on ecosystem function. Depending on the morphological, phenological, and physiological traits in response to environmental changes, different plant species differing in their ability to capture, store and input C and N to the soil, are a major driver of C and N storage in terrestrial ecosystems (Knops et al., 2002; Cortez et al., 2007; Cornwell et al., 2008; Carrera et al., 2009; Conti and Díaz, 2013). Grasslands cover approximately 40% of the earth’s land area and store a substantial amount of carbon (C) and nitrogen (N) in the terrestrial ecosystem (De Deyn et al., 2008). However, the body of empirical evidence which focus on the relationships between plant functional diversity and C or N storage in natural grassland ecosystems is still lacking (Cavanaugh et al., 2014).

Plant functional traits affect ecosystem properties via their effects on plant growth, tissue quality, and resource use (Lienin and Kleyer, 2012; Lavorel, 2013). Leaf dry matter content (LDMC), specific leaf area (SLA), and leaf nitrogen concentration (LNC), are three important traits of living leaves related to their structural properties and tissue quality, which affect the plant growth rate and litter quality (Kazakou et al., 2006; Cortez et al., 2007; Dechaine et al., 2014). Plant leaf with a high SLA is associated with high C input through high photosynthetic N use efficiency (Feng et al., 2008). Leaf traits with low values of the LNC and large values of the LDMC is referred to as the conservative resource use strategy, which leads to more C and N accumulation in standing biomass and lower C and N releases in litter decomposition (Kazakou et al., 2006; Poorter et al., 2009; Freschet et al., 2012; Milcu et al., 2014). More consistent studies have shown that the high-standing plants also offer more biomass and more C and N in the biomass and litter, thus contributing directly to C and N accumulation in soil (Lavorel and Grigulis, 2012; Lienin and Kleyer, 2012; Conti and Díaz, 2013). Thus, both the leaf traits and plant size have been linked to a series of ecosystem processes.

Plant functional diversity is reflected by the traits of the most abundant species (community-weighted mean, CWM) and the variety of functional trait values (Butterfield and Suding, 2013; Lavorel, 2013). Single-trait index of CWM showing the dominant trait value of most abundant species was defined as the abundance-weighted mean trait for a given community (Violle et al., 2007; Butterfield and Suding, 2013). The CWM effects may primarily be attributed to the biomass ratio hypothesis (Conti and Díaz, 2013; Lavorel, 2013). The biomass ratio hypothesis (Grime, 1998) proposes that ecosystem function is mainly driven by the plant traits of most abundant species which play key roles in the response of communities to habitat changes (Lepš et al., 2011; Bhaskar et al., 2014). CWM traits have been empirically proven as the main factor in determining the ecosystem C storage (Butterfield and Suding, 2013; Conti and Díaz, 2013; Milcu et al., 2014). However, recent studies have also reported that multi-trait index of functional dispersion (FDis) has a strong effect on ecosystem biomass and C stock (Mouillot et al., 2011; Ziter et al., 2013), supporting the niche complementarity hypothesis which proposes that the resource niches may be used more completely when a community is functionally more diverse (Tilman, 1997). Higher function dispersion reflects an increase in resource use complementarity among species in a plant community, which directly leads to an increased C storage in plant biomass (Ziter et al., 2013). Some studies have demonstrated that the mass ratio and niche-complementarity hypotheses are not necessarily mutually exclusive in explaining ecosystem function, and the combination of both of them has shown to be in operation in the natural ecosystems and increases the amount of explained variation (Schumacher and Roscher, 2009; Mouillot et al., 2011). So, examining the roles of both CWM traits and FDis in explaining ecosystem C and N storage can provide some insights into the mechanism of how plant functional diversity affects ecosystem function and properties.

Sandy grasslands which are characterized by sandy soils occupy a large percent of the natural vegetation in the semiarid regions of northern China (Li et al., 2009). Horqin sandy grassland is located in the semi-arid area of southeast Inner Mongolia and is notable for its severe desertification in northern China. However, as a result of annual precipitation ranging from 350 to 500 mm, mobile dunes (MDs) can be naturally restored to semi-fixed (SFD) or fixed dunes (FDs) after excluding grazing disturbance (Zhang et al., 2005; Liu et al., 2009; Zuo et al., 2015). Plant height, species richness and biomass increase with the vegetation restoration succession from sand pioneer plants in MDs to low shrub communities in SFDs, then toward annual herbaceous communities in FDs (Qiao et al., 2012; Zuo et al., 2014). The previous study has shown that sandy vegetation restoration may lead to the increase of C and N storage in the ecosystem, in which the total ecosystem C storage increased by 4.8 or 7.1 times from MDs to FDs or grasslands, and the corresponding N storage increased by 15.7 or 20.6 times (Zuo et al., 2015). However, little is known about the associations of plant functional diversity with ecosystem C or N storage in the sandy grassland restoration.

The objective of this study was to examine the relationships between different components of plant functional diversity and C or N storage in different ecosystem compartments along the restoration gradient of sandy grassland. We also attempted to identify the roles of CWM traits and FDis in predicting C and N storage in the sandy grassland restoration. Specifically, we tested the three hypotheses: (1) C and N storage in different ecosystem compartments could be predicted by the trait values of the most abundant species; (2) plant community dominated by plants with a higher investment in structure (such as high values of plant height) has higher C and N storage in standing biomass, litter and soil; and (3) the higher FDis correlates with the higher C and N storage in biomass and litter, thus leading to the higher ecosystem C and N storage in the sandy grassland restoration.

## Materials and Methods

### Site Description and Sampling Design

The study was carried out in southwest of Horqin Sandy Land (42°55′ N, 120°42′ E; 360 m elevation), Inner Mongolia, Northern China. The climate is a continental semiarid, with a warm summer and a very cold winter. The mean annual temperature is around 7.5°C, and the mean annual precipitation is 325.14 mm (data from the year 1991 to 2011 in Naiman Desertification Research Station), with 75% of the total in the growing season of the months of June to September. Sandy grasslands in this area are covered with native grasses, forbs and shrubs. The annual forb, *Agriophyllum squarrosum*, as a sand pioneer plant, is a dominant species on MDs (vegetation cover is less than 10%). SFDs (10–60%) were dominated by *Artemisia halodendron* shrub. FDs (>60%) were dominated by annual forb, *Artemisia scoparia* and perennial grass, *Cleistogenes squarrosa*. Grasslands (>60%) were dominated by annual forb, *A. scoparia* and perennial grasses, *Phragmites communis* and *Pennisetum centrasiaticum*.

Within the study area, we selected 24 sites from four typical habitats (six replicate sites per habitat) along a restoration gradient of sandy grassland, including MD, SFD, FD, and grassland (G). Study sites were the same as those used in our previous work (Zuo et al., 2015). SFD and FDs were derived from MDs by fencing for grazing exclusion approximately from 1995 and 1980, respectively. Grasslands were excluded from grazing by fencing in 1996. At each site, we established a 20 m × 20 m plot (replicate) in an area with flat topography (slope < 5°). Then we set up five 1 m × 1 m quadrats at the four corners and at the center in each plot to carry out vegetation survey, plant traits, and soil sampling. A description of the vegetation characteristics and soil properties in different habitat types is shown in Supplementary Table S1.

### Carbon and Nitrogen Storage

In each 1 m × 1 m quadrat, we recorded the species number and plant cover. Above-ground biomass of each species was harvested during the peak of the growing season, and then litter was collected within each quadrat. After the litter layer was removed in each quadrat, roots were taken from three core locations at four depth increments: 0 to 10, 10 to 20, 20 to 40, and 40 to 60 cm using an 8 cm-diameter soil auger. In calculating the soil bulk density, four soil samples were collected from the same four depths in each quadrat using a soil auger equipped with a stainless-steel cylinder (5 cm in both diameter and height). Concurrently, we also collected three random soil samples at each depth within the quadrat using a 3 cm-diameter soil auger and they were combined to form a composite sample for C and N content analysis.

Roots were washed and handpicked over a 1-mm screen to remove soil, pebbles and debris. The aboveground biomass, litter and roots were dried at a temperature of 60°C for 48 h and were ground using a mill (Pulverisette 14, German). Soil samples were air-dried and hand-sieved through a 2-mm screen to remove roots and other debris, and were ground using a soil grinding bowl and rod. Plant and soil samples were analyzed by an elemental analyzer (vario Macro cube, Elementar, Germany) to determine the C and N content. Soil C and N content at different depths were converted to g C and N m^2^ by the corresponding soil bulk density. The C and N content in plant, litter and root were also converted to g C and N m^-2^ by the corresponding biomass. We quantified C and N storage in the standing aboveground biomass (AGB), aboveground litter (AL), belowground root (BGR), and soil down to a depth of 60 cm, and then calculated the total ecosystem C (TEC) and N (TEN) storage. All C and N storage in the different ecosystem compartments are presented in Supplementary Table S2.

### Plant Functional Traits

To estimate the trait values of different plant communities along the whole gradient, we selected the 34 most abundant species (total 45 species) which accounted for 90% of the total biomass in the sandy grasslands, including forbs, grasses, and shrubs. For species richness, the forb life group dominated the four habitats (Supplementary Table S3). Species abundance thus corresponds to the relative biomass of each species to the total biomass in the plot. We selected six key traits that are known to affect ecosystem function (De Deyn et al., 2008; Lepš et al., 2011; Conti and Díaz, 2013), including the plant height, SLA, LDMC, leaf carbon content (LCC), leaf nitrogen content (LNC), and root density (RD). The first five traits of 5–10 plants per species in each quadrat were measured using standard methods (Cornelissen et al., 2003; Kichenin et al., 2013). RD related to root biomass was calculated down to a depth of 60 cm (Butterfield and Suding, 2013). The functional trait values of 34 species included in the study area are shown in Supplementary Table S4.

### Function Diversity Measurement

We used two different components of functional diversity: the dominant trait values and the variety of trait values (Díaz et al., 2007). The CWM expressing the dominant trait value in each plot was calculated as the abundance-weighted mean trait value for a community (Violle et al., 2007; Butterfield and Suding, 2013): CWM (trait_X_) = Σ*p*_i_*x*_i_, where CWM (trait_X_) is the CWM for *X* trait, *p*_i_ is the relative biomass of the *i*-th species in the community, and *x*_i_ is the trait value of the *i*-th species. Functional divergence (FDvar) was calculated to express the single-trait divergence for a community (Conti and Díaz, 2013). We also calculated FDis in order to express the multiple traits dispersion within the functional volume of the community (Laliberté and Legendre, 2010). FDis may be regarded as a surrogate measure of functional richness and FDvar (Schleicher et al., 2011). We chose the three indices of CWM, FDvar, and FDis in this study, because they had also been successfully applied to empirical studies with similar objectives (Butterfield and Suding, 2013; Conti and Díaz, 2013; Spasojevic et al., 2014). All functional diversity indices were calculated using the statistical package FDiversity v. 2011(Casanoves et al., 2011).

### Relationships Between Function Diversity and C and N Storage

We first of all, used the correlation analysis to examine whether C and N storage in AGB, AL, root, and soil was related to CWM, FDvar, and FDis. Then, we used the linear (for example, simple linear regression and log-transformation linear regression) and non-linear models (for example, power and exponential models) to quantify the potential relationships between the total ecosystem C and N storage and the components of functional diversity. Based on the explanation variation (*R*^2^) of models, we found that simple linear regressions were straight in showing how the components of functional diversity explained the C and N storage in the total ecosystem. Finally, all the variables that significantly explained C and N storage in the analyses of correlation and simple linear regression were included in the multiple linear regression analyses, following a stepwise model selection procedure, in order to select the best predictors of C and N storage in different ecosystem compartments (Conti and Díaz, 2013). All statistical analyses were performed using SPSS (version 16.0). Abbreviations of habitat, functional trait, functional diversity index, carbon storage and nitrogen storage are shown in Supplementary Table S5.

## Results

Habitat changes, following sandy grassland restoration had significant effects on six CWM traits, the functional trait variety of LCC and the FDis (**Table 1**, *P* < 0.05). CWM of plant height and RD increased along the restoration gradient of the sandy grassland. CWM of LCC increased from MD to FD. CWMs of SLA and LDMC in SFD were lower than in the other three habitats and CWM of LNC in MD was higher than in the other three habitats. FDvar of LCC in MD and SFD was higher than in FD and grassland. FDis in grassland was higher than in the three dune habitats, but there was no difference among the three dune habitats. So, single- and multi-trait functional indices had strong responses to habitat changes in the sandy grassland restoration.

**Table 1:** Changes of functional diversity components at four habitats of sandy grassland (Mean ± SE, *n* = 6).

	MD	SFD	FD	G	F	*P*
**Community weighted means (CWM)**						
Height (cm)	9.23 ± 0.99^a^	24.95 ± 2.63^b^	43.12 ± 2.11^c^	64.80 ± 5.81^d^	49.66	<0.001
SLA (m–^2^ kg^-1^)	16.83 ± 1.58^a^	12.93 ± 0.85^b^	19.12 ± 0.56^a^	19.48 ± 0.77^a^	8.82	<0.01
LDMC (g kg^-1^)	261.12 ± 9.76^a^	207.68 ± 9.43^b^	270.42 ± 11.33^a^	279.98 ± 10.22^a^	10.03	<0.001
LCC (%)	39.38 ± 0.55^a^	43.52 ± 0.72^b^	45.25 ± 0.14^c^	44.72 ± 0.25^bc^	31.52	<0.001
LNC (%)	2.75 ± 0.12^a^	2.23 ± 0.06^b^	2.32 ± 0.04^b^	2.27 ± 0.08^b^	8.64	<0.01
RD(g m^-3^)	3.19 ± 1.07^a^	19.50 ± 3.68^b^	13.54 ± 2.42^b^	27.55 ± 4.80^c^	9.67	<0.001
**Single-trait functional divergence indices (FDvar)**						
Height (cm)	0.23 ± 0.1^a^	0.48 ± 0.04^a^	0.46 ± 0.06^a^	0.43 ± 0.08^a^	2.32	0.107
SLA (m^-2^ kg^-1^)	0.11 ± 0.05^a^	0.13 ± 0.03^a^	0.09 ± 0.04^a^	0.22 ± 0.1^a^	0.88	0.47
LDMC (g kg^-1^)	0.12 ± 0.03^a^	0.08 ± 0.03^a^	0.09 ± 0.02^a^	0.16 ± 0.04^a^	1.05	0.391
LCC (%)	0.013 ± 0.010^ab^	0.018 ± 0.001^b^	0.002 ± 0.001^a^	0.002 ± 0.001^a^	3.58	<0.05
LNC (%)	0.09 ± 0.03^a^	0.09 ± 0.05^a^	0.04 ± 0.01^a^	0.06 ± 0.03^a^	0.45	0.723
**Multi-trait FDvar index(Log transformation)**						
FDis	0.06 ± 0.02^a^	0.09 ± 0.01^a^	0.06 ± 0.011^a^	0.12 ± 0.02^b^	4.04	<0.05

*MD, Mobile dune; SFD, Semi-fixed dune; FD, Fixed dune; G, Grassland; specific leaf area (SLA), leaf dry matter content (LDMC), leaf carbon content (LCC), leaf nitrogen content (LNC) and RD(0–60 cm), root density. Different letters in from mean values indicate statistical difference among different habitats at *P* < 0.05.*

A number of individual components of functional diversity significantly correlated with the C and N storage in different ecosystem compartments (**Table 2**). CWMs of height, LCC and RD were significantly and positively correlated with the C and N storage in AGB, AL, BGR, and soil, while the CWM of LNC was significantly and negatively correlated with them. CWMs of SLA and LDMC were significantly and positively correlated with soil C and N storage. FDvar of SLA was significantly and positively correlated with the C and N storage in BGR, and FDvar of LCC was significantly and negatively correlated with the C and N storage in AGB and soil. FDis was significantly and positively correlated with the C and N storage in AL and soil, as well as the C storage in BGR (**Table 2**).

**Table 2:** Correlation coefficients between functional diversity components and C, N storage in different ecosystem compartments (*n* = 24).

	Height	SLA	LDMC	LCC	LNC	RD	FDvar Height	FDvar SLA	FDvar LDMC	FDvar LCC	FDvar LNC	FDis
SLA	0.40											
LDMC	0.36	0.37										
LCC	0.76^**^	0.08	0.10									
LNC	-0.47^*^	-0.04	0.11	-0.58^**^								
RD	0.58^**^	-0.08	0.12	0.57^**^	-0.59^**^							
FDvar Height	0.19	0.09	0.12	0.31	-0.49^*^	0.38						
FDvar SLA	0.10	0.09	0.23	-0.05	-0.35	0.53^**^	0.37					
FDvar LDMC	0.08	0.41^*^	0.12	-0.16	0.05	0.23	0.31	0.59^**^				
FDvar LCC	-0.50^*^	-0.40	-0.22	-0.42^*^	0.08	-0.15	0.25	0.11	0.21			
FDvar LNC	-0.20	-0.15	-0.02	-0.14	-0.18	0.10	0.39	0.42^*^	0.35	0.37		
FDis	0.29	0.30	0.00	0.09	-0.43^*^	0.52^**^	0.69^**^	0.75^**^	0.55^**^	0.16	0.41^*^	
**Carbon storage**								
AGB	0.96^**^	0.25	0.21	0.82^**^	-0.58^**^	0.62^**^	0.25	0.06	0.01	-0.43^*^	-0.20	0.28
AL	0.84^**^	0.23	0.15	0.64^**^	-0.43^*^	0.79^**^	0.22	0.36	0.26	-0.36	-0.09	0.49^*^
BGR	0.54^**^	-0.13	0.07	0.55^**^	-0.62^**^	0.99^**^	0.39	0.51^*^	0.19	-0.11	0.14	0.50^*^
Soil	0.84^**^	0.59^**^	0.52^**^	0.65^**^	-0.44^*^	0.52^**^	0.37	0.31	0.29	-0.48^*^	-0.12	0.47^*^
**Nitrogen storage**								
AGB	0.97^**^	0.38	0.33	0.77^**^	-0.47^*^	0.55^**^	0.20	0.10	0.11	-0.48^*^	-0.22	0.27
AL	0.82^**^	0.32	0.27	0.58^**^	-0.41^*^	0.78^**^	0.21	0.40	0.30	-0.38	-0.05	0.47^*^
BGR	0.58^**^	-0.07	0.12	0.57^**^	-0.54^**^	0.93^**^	0.30	0.46^*^	0.19	-0.14	0.09	0.37
Soil	0.82^**^	0.62^**^	0.54^**^	0.63^**^	-0.44^*^	0.47^*^	0.37	0.31	0.29	-0.49^*^	-0.10	0.44^*^

*SLA, specific leaf area; LDMC, leaf dry matter content, LCC, leaf carbon content, LNC, leaf nitrogen content; RD(0–60 cm), root density; AGB, aboveground standing biomass; AL, aboveground litter mass; BGR, belowground root biomass(0–60 cm); ^∗^*P* < 0.05; ^∗∗^*P* < 0.01.*

We also found that FDis contained a great deal of information from their single-trait and tended to represent different dimensions of functional composition (**Table 2**). FDis was significantly and positively correlated with the CWM of RD and FDvar of height, SLA, LDMC, and LNC, and was significantly and negatively correlated with the CWM of LNC. Within single trait indices, the CWM of height was significantly and positively correlated with the CWMs of LCC and RD, and was significantly and negatively correlated with the CWM of LNC and FDvar of LCC. RD was significantly and positively correlated with the FDvar of SLA. CWM of LCC was significantly and negatively correlated with CWM of LNC and FDvar of LCC. CWM of LNC was significantly and negatively correlated with the FDvar of height.

**FIGURE 1:**
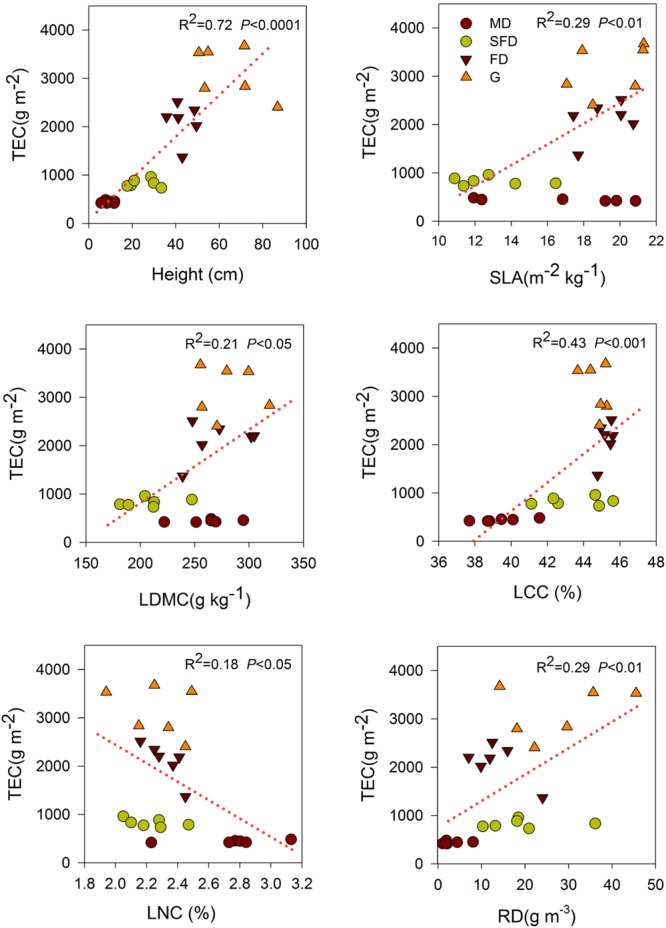
**Simple linear regression analyses between total ecosystem C storage and community-weighted mean (CWM) of functional traits.** MD, Mobile dune; SFD, Semi-fixed dune; FD, Fixed dune; G, grassland; specific leaf area (SLA), leaf dry matter content (LDMC), leaf carbon content (LCC), leaf nitrogen content (LNC), and root density (RD).

**FIGURE 2:**
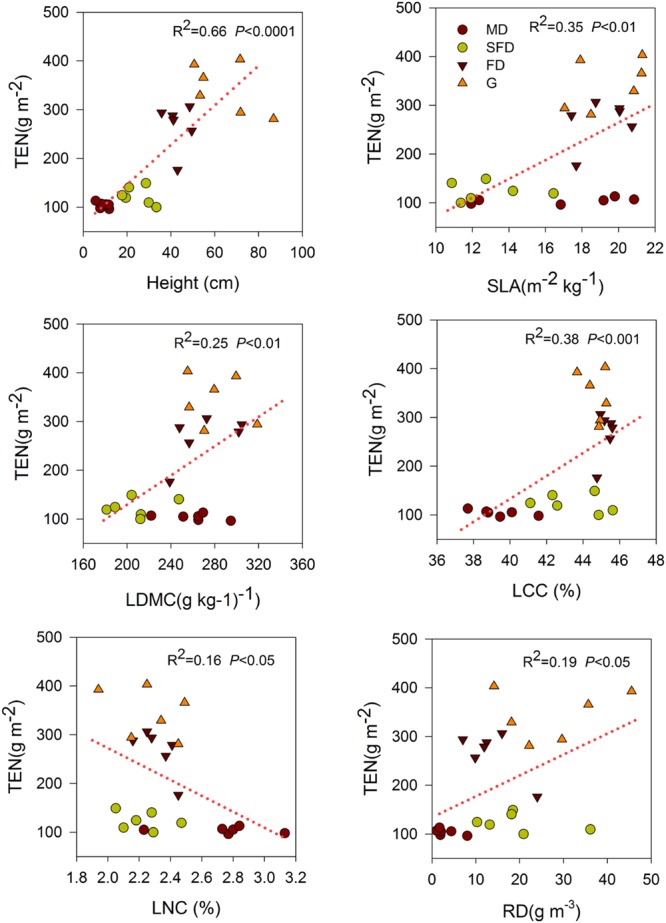
**Simple linear regression analyses between total ecosystem N storage and CWM of functional traits.** MD, Mobile dune; SFD, Semi-fixed dune; FD, Fixed dune; G, grassland; SLA, LDMC, LCC, LNC, and RD.

**FIGURE 3:**
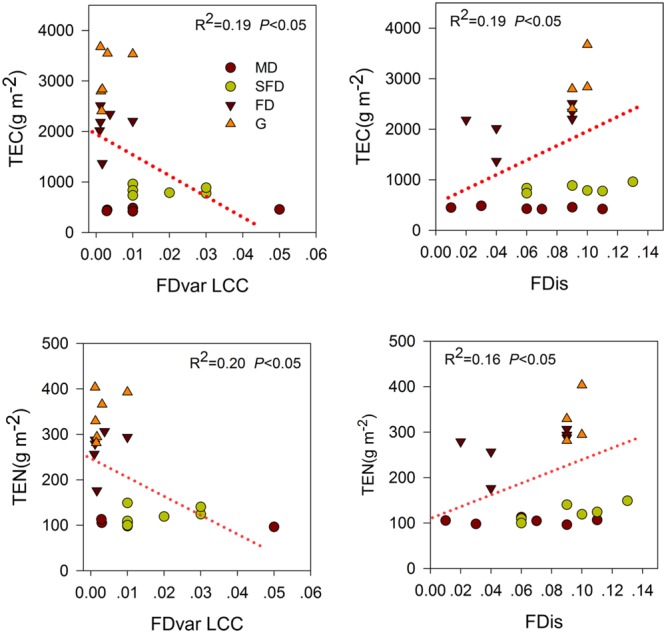
**Simple linear regression analyses between ecosystem C and N storage and single-trait functional divergence (FDvar LCC), multiple traits dispersion (FDis).** MD, Mobile dune; SFD, Semi-fixed dune; FD, Fixed dune; G, grassland.

Single-trait functional diversity significantly explained the total ecosystem C and N storage (**Figures 1** and **2**). CWMs of plant height, SLA, LDMC, LCC, and RD were significantly and positively associated with TEC and TEN storage, while the CWM of LNC was significantly and negatively associated with them. FDvar of LCC was significantly and negatively associated with TEC and TEN storage (**Figure 3**). FDis was also significantly and negatively associated with TEC and TEN storage (**Figure 3**).

When all functional diversity components that significantly explained C and N storage in different ecosystem compartments were combined in the multiple linear regression models, the single-trait indices associated with plant height and leaf were retained in the most final models (**Table 3**). C storage in AGB was best explained positively by the CWM of height and negatively by the CWM of LNC. N storage in AGB was best explained positively by the CWM of height. C and N storage in AL was best explained positively by the CWMs of height and FDis. C and N storage in BGR was best explained negatively by the CWM of RD. Soil C and N storage was best explained positively by the CWMs of height and SLA. Finally, TEC storage was best explained positively by FDis and the CWMs of both height and LDMC. TEN storage was best explained positively by the CWMs of height and SLA.

**Table 3:** Final models of stepwise regression analyses between the magnitude of C and N stocks and functional diversity components, using a stepwise ascending procedure.

	Model	Predictor variables	Slope	*P*	*n*	*R*^2^
**Carbon storage**						
Aboveground standing biomass carbon (AGBC)	AGBC = 45.14 + 1.30 Height – 18.72 LNC	Model Height	+	<0.0001 <0.0001	24	0.94
		LNC	–	0.013		
Aboveground litter carbon (ALC)	ALC = –14.54 + 0.91 Height + 168.20 FDis	Model Height	+	<0.0001 <0.0001	24	0.75
		Fdis	+	0.024		
Belowground root carbon (BGRC)	BGRC = –2.04 + 3.66 RD	Model RD	+	<0.0001 <0.0001	24	0.98
Soil total carbon (STC)	STC = –1265.81 + 33.73 Height + 91.33 SLA	Model Height	+	<0.0001 <0.0001	24	0.76
		SLA	+	0.013		
Total ecosystem carbon (TEC)	TEC = –2154.62 + 34.38 Height + 7456.74 FDis + 7.59 LDMC	Model Height FDis	+ +	<0.0001 <0.0001 0.006	24	0.82
		LDMC	+	0.017		
**Nitrogen storage**						
Aboveground standing biomass nitrogen (AGBN)	AGBN = –0.32 + 0.06 Height	Model Height	+	<0.0001 <0.0001	24	0.94
Aboveground litter nitrogen (ALN)	ALN = –0.47 + 0.02 Height + 168.20 FDis	Model Height	+	<0.0001 <0.0001	24	0.70
		FDis	+	0.04		
Belowground root nitrogen (BGRN)	BGRN = 0.10 + 0.07 RD	Model RD	+	<0.0001 <0.0001	24	0.87
Soil total nitrogen (STN)	STN = –82.91 + 3.17 Height + 10.33 SLA	Model Height	+	<0.0001 <0.0001	24	0.75
		SLA	+	0.007		
Total ecosystem nitrogen (TEN)	TEN = –81.56 + 3.28 Height + 10.23 SLA	Model Height	+	<0.0001 <0.0001	24	0.75
		SLA	+	0.007		

*All multiple regression models were statistically significant (*P* < 0.05). *R*^2^, regression adjusted coefficient; N, number of plots in the analyses; slope and *P* refer to individual predictor variables in the final model; Height, SLA, LDMC, LCC, and LNC calculated from the community-weighted mean (CMW); RD, root density; FDis, multi-trait functional index.*

## Discussion

To the best of our knowledge, this is the first study to empirically test the associations between different components of functional diversity and the C and N storage in sandy grassland ecosystems. Our results had shown how plant functional diversity explained ecosystem C and N stocks in sandy grassland restoration, which could be extensively discussed in the trait theory of functional diversity effects on ecosystem function. Our results suggest that the dominant species’ traits reflected by CWM are the important predictors of ecosystem C and N storage; however, FDis is only important for ecosystem C storage in sandy grassland restoration. A combined effect of the CWM traits and FDis is the most important explanation for ecosystem C storage in the sandy grassland restoration, supporting the finding that the mass ratio and niche-complementarity hypotheses in combination may explain ecosystem function and properties (Schumacher and Roscher, 2009; Mouillot et al., 2011).

In the final models, C and N storage in most of the ecosystem compartments could be best explained by the CWM traits, thus supporting the first hypothesis that the trait values of the most abundant species could predict C and N storage in accordance with the mass ratio model. This is also consistent with other studies that ecosystem function and properties are primarily driven by the CWM traits (Schumacher and Roscher, 2009; Ruiz-Jaen and Potvin, 2011; Conti and Díaz, 2013). The previous study has shown that plant community-level traits may be caused by changes in the dominant species with different trait values, changes in trait values within a species or a combination of these two (Lepš et al., 2011). In our case, the relative differences between CWM traits affect C and N storage more heavily than the trait dispersion within-community, due to the restoration succession of sandy grassland vegetation. This is particularly relevant when the community-level trait values are weighted by the abundance of each species, and therefore emphasizes the effect of traits of the dominant species on ecosystem C and N storage (Díaz et al., 2007; Conti and Díaz, 2013).

Not surprisingly, we found that the CWM of plant height was included in the final models as an important factor in explaining C and N storage in the aboveground plant, litter and soil, as well as total ecosystem, which supported the second hypothesis. This is in agreement with other findings that the CWM of plant height is one of the best predictors of ecosystem C storage (Garnier et al., 2004; Lavorel and Grigulis, 2012; Conti and Díaz, 2013). Our results have suggested that the habitat where the sandy grassland restoration has led to the local dominance of the relatively higher plants is associated with more C and N storage in the vegetation, litter, and soil. This may be based on the fact that plant communities with higher plant height can lead to the higher plant biomass, thus contributing to promote soil C and N storage because of inputs of abundant litter at senescence in the sandy grassland restoration.

We also found that both C and N storage in soil or total ecosystem were positively correlated with the CWMs of SLA and LDMC in sandy grassland restoration (**Figure 1**, **Table 2**). However, in the final models, CWM of SLA was one of the two best predictors of soil C and N storage, and the CWM of LDMC was one of the most important predictors of the total ecosystem C storage. This is conceptually consistent with the previous studies that leaf traits are known to have an exploitative strategy with fast nutrient acquisition, fast growth and high productivity, and thus are expected to be a significant predictor of C and N storage (Wright et al., 2004; Poorter et al., 2009). Our previous studies showed that C and N storage in plant, litter, and soil increased with vegetation restoration succession from sand pioneer plants in MDs to low shrub communities in SFDs, then toward annual herbaceous communities in FDs and grasslands (Zuo et al., 2009, 2015). So, these results suggest that soil C and N storage are likely to be linked with the dominant leaf traits of the most abundant species in the vegetation restoration succession of sandy grassland. Our results further suggest that what matters the most to soil C and N storage is the relative abundance of plants with high-standing and larger SLA in the sandy grassland ecosystems.

The variety of leaf C content values were expressed as the CWM of LCC and FDvar of LCC which separately had a positive and negative association with C or N storage in the above-ground standing biomass, soil, and total ecosystem in the analyses of correlation and regression (**Table 2**, **Figure 3**), while none of them was retained in the final models. Although the high C concentration in leaf may increase the leaf mass, the litter mass is determined by plant growth or plant biomass in ecosystem. In our study, this was also likely to relate to the fact that habitats dominated by the higher plants with a high C content in leaf had a large litter mass following sandy grassland restoration. Thus, the direct effects of LCC on C and N storage might be reflected by plant height in the final models, as indicated by the high correlation between them (*r* = 0.76, **Table 2**). The significantly negative relationship between the CWM of LCC and FDvar of LCC showed that CWM of LCC increased, while the variety of community-level LCC decreased, suggesting that the increase of LCC in most species enhances plant biomass or litter mass and thus favors soil C and N storage in the sandy grassland restoration.

Multi-trait dispersion, as expressed by FDis, was positively associated with the total ecosystem C storage in the final model. Litter C and N storage were also positively associated with FDis. These results partly supported the third hypothesis that plant community in higher FDis had a higher C storage in litter and total ecosystem in the sandy grassland restoration. This provides an evidence to support the niche-complementarity hypothesis (Tilman, 1997), suggesting that plant FDis has also an important role to play in the ecosystem C stock (Mouillot et al., 2011; Ziter et al., 2013). This complementarity effect implies that the total ecosystem C storage is also likely to depend on the functional characteristics of the constituent species in sandy grasslands. Following changes in dominant species and increase of species richness in the sandy grassland restoration, niche partitioning or facilitation among species can lead to plant communities with high functional trait dispersion. The high functional trait dispersion in the community is likely to promote litter production or accumulation, thus contributing to the total ecosystem C storage. In our study, FDis is at least a good predictor of the total ecosystem C storage as the best component of CWM traits (Mouillot et al., 2011).

Functional dispersion had a significant positive association with N storage in litter, soil and total ecosystem in the correlation and regression analyses (**Figure 3**, **Table 2**). However, N storage in most components of the ecosystem was best explained by the CWM traits in the final models. Previous studies have shown that N storage of these components in the ecosystem was mainly determined by species composition (Epstein et al., 1998; Carrera et al., 2009), litter quality and decomposition (Shaw and Harte, 2001; Knops et al., 2007). Therefore, the association between the functional diversity and the total ecosystem N storage may primarily be explained by the biomass ratio hypothesis rather than by the niche complementarity hypothesis.

## Conclusion

Our results have clearly illustrated the important roles of CWM traits and FDis in explaining the C and N storage in sandy grassland restoration. Our findings suggest that the two major components of plant functional diversity: the dominant trait values and the function dispersion in the community, can contribute to explaining C storage at the ecosystem level. The dominant traits with strong explanatory power over C and N storage were plant height at community-level in a sandy grassland ecosystem. Community-level functional traits with investments in exploitative strategy linked to plant height and SLA were proven to be important predictors of soil C and N storage, as well as the total ecosystem N storage. The trait values of the most abundant species in plant communities may best explain the ecosystem C and N pools in the sandy grassland restoration, which further reinforces the mass ratio hypothesis. Our finding also demonstrates that the dispersion of functional traits affects the litter C and N storage as well as the total ecosystem C storage, thus supporting that the niche complementarity also plays an important role in the C storage capacity in sandy grassland ecosystems. These results suggest that the mass ratio and niche complementarity models are not mutually exclusive: they both play an important role in the ecosystem C storage in the sandy grassland restoration. Therefore, changes in dominant species following habitat change deserve the most attention when C and N storage is maintained in sandy grassland ecosystems. At the same time, efforts need to be made to modify the functional structure of communities by selecting plants with wide niche or trait ranges in order to increase the C storage in sandy grassland ecosystems.

## Author Contributions

XZ designed the research, collected and analyzed the data, and drafted the paper. XZ collected the data and did some lab analysis. PL assisted with data collection. XZ revised English language and contributed to paper writing. JZ assisted with data collection. SW assisted with data collection. XY assisted with data collection. All authors depending on their contribution for this paper have approved the final article to publish.

## Conflict of Interest Statement

The authors declare that the research was conducted in the absence of any commercial or financial relationships that could be construed as a potential conflict of interest.
