# Supplementary material for: Testing Associations of Plant Functional Diversity with Carbon and Nitrogen Storage along a Restoration Gradient of Sandy Grassland
Source: Front Plant Sci. 2016 Feb 19;7:189. doi: 10.3389/fpls.2016.00189 (PMC4759253; doi:10.3389/fpls.2016.00189)
Supplement: Supplementary file 1 [file Table_1.DOCX]

Table S1. Vegetation characteristics and soil properties at four habitats of sandy grassland (Mean ± SE, N=6).

|  | MD | SFD | FD | G | F | *P* |
| --- | --- | --- | --- | --- | --- | --- |
| **Vegetation characteristics** |  |  |  |  |  |  |
| Species richness | 3.33±0.49^a^ | 10.83±0.75^b^ | 17.50±1.12^c^ | 13.67±1.23^d^ | 40.27 | < 0.001 |
| Biomass( g m^-2^) | 2.20±0.39^a^ | 98.10±7.27^b^ | 131.41±8.20^c^ | 190.38±19.15^d^ | 50.93 | < 0.001 |
| **Soil properties（0-10cm）** |  |  |  |  |  |  |
| C (g kg^-1^) | 0.43±0.01^a^ | 1.10±0.08^b^ | 3.94±0.19^c^ | 5.17±0.29^d^ | 160.50 | < 0.001 |
| N (g kg^-1^) | 0.11±0.01^a^ | 0.15±0.01^a^ | 0.47±0.02^b^ | 0.61±0.03^c^ | 122.00 | < 0.001 |
| C/N | 4.23±0.43^a^ | 7.56±0.30^b^ | 8.48±0.21^c^ | 8.48±0.12^d^ | 49.16 | < 0.001 |
| pH | 7.39±0.02^a^ | 7.50±0.04^a^ | 8.01±0.10^b^ | 8.47±0.06^c^ | 58.02 | < 0.001 |
| Bulk density (g cm^-3^) | 1.62±0.02^a^ | 1.57±0.01^a^ | 1.50±0.02^b^ | 1.33±0.04^c^ | 26.38 | < 0.001 |
| Electricity conductivity (μs cm^-1^) | 7.83±0.49^a^ | 11.23±0.98^a^ | 20.93±1.71^b^ | 30.00±3.41^c^ | 25.47 | < 0.001 |
| Coarse sand (2-0.25 mm, %) | 60.68±2.03^a^ | 47.01±1.55^b^ | 43.65±1.65^b^ | 21.33±4.72^c^ | 33.82 | < 0.001 |
| Fine sand (0.25-0.1 mm, %) | 37.49±2.12^ac^ | 49.73±1.31^b^ | 42.22±3.16^a^ | 35.22±2.35^c^ | 7.57 | < 0.01 |
| Very fine sand (0.1-0.05 mm, %) | 1.48±0.09^a^ | 1.85±0.27^a^ | 5.62±1.32^a^ | 31.36±5.43^b^ | 26.15 | < 0.001 |
| Silt + Clay (<0.05 mm, %) | 0.35±0.03^a^ | 1.41±0.23^a^ | 8.51±0.61^b^ | 12.09±1.77^c^ | 35.90 | < 0.001 |
| Soil water content (%) | 2.86±0.17^a^ | 2.60±0.28^a^ | 3.36±0.51^a^ | 5.01±0.53^b^ | 7.23 | < 0.01 |

MD, Mobile dune; SFD, Semi-fixed dune; FD, Fixed dune; G, Grassland; Different letters in from mean values indicate statistical difference among different habitats at *P*<0.05.
