# Supplementary material for: Testing Associations of Plant Functional Diversity with Carbon and Nitrogen Storage along a Restoration Gradient of Sandy Grassland
Source: Front Plant Sci. 2016 Feb 19;7:189. doi: 10.3389/fpls.2016.00189 (PMC4759253; doi:10.3389/fpls.2016.00189)
Supplement: Supplementary file 2 [file Table_2.DOCX]

Table S2. Carbon (C) and nitrogen (N) storage in different ecosystem compartments at four habitats of sandy grassland (Mean ± SE, N=6).

|  | MD | SFD | FD | G | F | *P* |
| --- | --- | --- | --- | --- | --- | --- |
| **Carbon storage (g m^-2^)** |  |  |  |  |  |  |
| Aboveground standing biomass | 0.85±0.15^a^ | 43.17±3.72^b^ | 57.33±3.86^c^ | 84.77±8.79^d^ | 46.22 | < 0.001 |
| Aboveground Litter | 1.02±0.42^a^ | 29.74±4.53^b^ | 29.49±7.19^c^ | 68.15±5.27^d^ | 30.34 | < 0.001 |
| Belowground Root (0-60 cm) | 9.77±3.19^a^ | 73.53±14.75^bc^ | 45.77±9.39^ac^ | 96.3±17.66^b^ | 8.86 | < 0.01 |
| Soil (0-60 cm) | 431.44±9.66^a^ | 683.87±36.16^a^ | 1973.47±176.60^b^ | 2880.96±212.09^c^ | 67.71 | < 0.001 |
| Total ecosystem | 443.07±10.32^a^ | 830.32±33.59^a^ | 2106.04±162.45^b^ | 3130.17±212.55^c^ | 82.88 | < 0.001 |
| **Nitrogen storage (g m^-2^)** |  |  |  |  |  |  |
| Aboveground standing biomass | 0.06±0.01^a^ | 1.12±0.07^b^ | 2.23±0.17^c^ | 3.15±0.37^d^ | 43.13 | < 0.001 |
| Aboveground Litter | 0.03±0.01^a^ | 0.50±0.08^b^ | 0.76±0.24^b^ | 1.77±0.13^c^ | 26.89 | < 0.001 |
| Belowground Root (0-60 cm) | 0.28±0.11^a^ | 1.36±0.25^b^ | 1.27±0.35^b^ | 1.93±0.34^b^ | 6.00 | < 0.01 |
| Soil (0-60 cm) | 103.79±2.61^a^ | 120.83±7.68^a^ | 262.53±19.78^b^ | 337.42±21.01^c^ | 56.63 | < 0.001 |
| Total ecosystem | 104.15±2.51^a^ | 123.82±7.56^a^ | 266.78±19.31^b^ | 344.27±20.83^c^ | 61.10 | < 0.001 |

MD, Mobile dune; SFD, Semi-fixed dune; FD, Fixed dune; G, Grassland; Different letters in from mean values indicate statistical difference among different habitats at *P*<0.05.
