# Supplementary material for: Testing Associations of Plant Functional Diversity with Carbon and Nitrogen Storage along a Restoration Gradient of Sandy Grassland
Source: Front Plant Sci. 2016 Feb 19;7:189. doi: 10.3389/fpls.2016.00189 (PMC4759253; doi:10.3389/fpls.2016.00189)
Supplement: Supplementary file 3 [file Table_3.DOCX]

Table S3. Changes of species number, proportion of species number and dominance values (DV) of the forb, grass and shrub at four habitats of sandy grassland.

|  | Forb | | | Grass | | | Shrub | | |
| --- | --- | --- | --- | --- | --- | --- | --- | --- | --- |
|  | Species number | Proportion  (%) | Dominant value (%) | Species number | Proportion  (%) | Dominant value  (%) | Species number | Proportion  (%) | Dominant value  (%) |
| MD | 3 | 42.86 | 60.68 | 3 | 42.86 | 37.64 | 1 | 14.29 | 1.68 |
| SFD | 10 | 66.67 | 45.60 | 3 | 20.00 | 12.79 | 2 | 13.33 | 41.19 |
| FD | 13 | 56.52 | 77.40 | 7 | 30.43 | 11.33 | 3 | 13.04 | 6.60 |
| G | 16 | 69.57 | 65.74 | 6 | 26.09 | 28.49 | 1 | 4.35 | 4.97 |

MD, Mobile dune; SFD, Semi-fixed dune; FD, Fixed dune; G, Grassland; The specie dominance (DV) in each habitat was calculated using the ordinary formula DV = (RW + RH + RC)/3, where RA is the relative biomass, RH is the relative height, and RC the relative cover of the species. RW, RH, and RC were all represented as percent values.
