# Supplementary material for: Testing Associations of Plant Functional Diversity with Carbon and Nitrogen Storage along a Restoration Gradient of Sandy Grassland
Source: Front Plant Sci. 2016 Feb 19;7:189. doi: 10.3389/fpls.2016.00189 (PMC4759253; doi:10.3389/fpls.2016.00189)
Supplement: Supplementary file 4 [file Table_4.DOCX]

Table S4. The most abundant species and their trait values used to calculate community functional indices. List of most abundant species and their trait values used to calculate community weighted mean (CWM) and functional divergence indices (FDvar and FDiv). H: height, SLA: specific leaf area, LDMC: leaf dry matter content, LCC: leaf carbon concentration, LNC: leaf nitrogen concentration, AF: Annual forb, AG: annual grass, PF: perennial forb, PG: perennial grass, S: shrub.

| Species | Life-form | H  (cm) | SlA  (m^-2^ kg^-1^) | LDMC  (g kg^-1^) | LCC  (%) | LNC  (%) |
| --- | --- | --- | --- | --- | --- | --- |
| *Corispermum macrocarpum* | AF | 7.44 | 12.74 | 215.22 | 37.35 | 2.37 |
| *Delphinium grandiflorum* | AF | 60.00 | 18.73 | 202.30 | 41.23 | 2.03 |
| *Artemisia sieversiana* | AF | 56.50 | 21.29 | 257.57 | 46.09 | 2.65 |
| *Euphorbia humifusa* | AF | 3.96 | 31.16 | 281.64 | 43.29 | 2.22 |
| *Cynanchum theisiodes* | AF | 9.23 | 20.77 | 221.18 | 45.45 | 2.88 |
| *Potentilla bifurca* | AF | 14.33 | 15.53 | 395.60 | 45.12 | 2.28 |
| *Artemisia scoparia* | AF | 58.27 | 20.95 | 252.95 | 45.30 | 2.33 |
| *Tribulus terretris* | AF | 1.81 | 33.99 | 244.46 | 41.60 | 3.33 |
| *Chenopodium acuminatum* | AF | 10.66 | 21.32 | 150.82 | 39.98 | 3.07 |
| *Sonchus oleraceus* | AF | 8.25 | 24.93 | 156.60 | 42.58 | 2.34 |
| *Ixeris denticulata* | AF | 15.75 | 16.05 | 217.03 | 46.06 | 2.22 |
| *Erodium stephanianum* | AF | 11.55 | 17.11 | 248.94 | 44.77 | 2.38 |
| *Agriophyllum squarrosum* | AF | 9.32 | 14.18 | 219.22 | 40.34 | 3.02 |
| *Echinops meliniturcz* | AF | 24.33 | 16.77 | 179.94 | 44.27 | 2.16 |
| *Bassia dasyphylla* | AF | 3.64 | 14.97 | 132.68 | 37.90 | 2.07 |
| *Caragana microphylla* | AF | 24.72 | 15.58 | 344.06 | 46.17 | 3.79 |
| *Salsola collina* | AF | 5.37 | 14.14 | 198.49 | 36.12 | 1.86 |
| *Setaria viridis* | AG | 9.05 | 23.71 | 292.19 | 40.83 | 1.98 |
| *Eragrostis pilosa* | AG | 6.67 | 28.68 | 357.90 | 40.80 | 1.99 |
| *Digitaria sanguinalis* | AG | 3.82 | 30.68 | 217.18 | 40.35 | 1.70 |
| *Tragus berteronianus* | AG | 1.92 | 32.63 | 332.13 | 43.46 | 2.17 |
| *Artemisia argyi* | PF | 59.00 | 16.48 | 297.00 | 47.42 | 1.76 |
| *Melissitus ruthenicus* | PF | 26.00 | 23.49 | 342.30 | 45.65 | 2.97 |
| *Allium mongolicum* | PF | 25.80 | 15.24 | 99.27 | 43.12 | 2.07 |
| *Gueldenstaedtia stenophylla* | PF | 6.00 | 16.64 | 325.50 | 44.82 | 3.26 |
| *Pennisetum centrasiaticum* | PG | 29.01 | 18.17 | 308.24 | 42.38 | 1.69 |
| *Cleistogenes squarrosa* | PG | 13.86 | 22.68 | 442.87 | 44.10 | 1.74 |
| *Leymus secalinus* | PG | 46.83 | 8.68 | 358.10 | 45.16 | 1.53 |
| *Phragmites communis* | PG | 30.20 | 11.78 | 366.31 | 42.74 | 2.46 |
| *Artemisia halodendron* | S | 32.23 | 11.69 | 214.73 | 46.40 | 2.34 |
| *Lespedeza davurica* | S | 13.54 | 13.59 | 400.87 | 46.66 | 2.43 |
| *Salix gordejevii* | S | 25.00 | 14.28 | 328.10 | 39.98 | 2.30 |
| *Artemisia frigida* | S | 38.75 | 13.96 | 347.50 | 47.25 | 2.73 |
| *Atraphaxis manshurica* | S | 45.34 | 7.75 | 333.80 | 39.98 | 2.40 |
