# Supplementary material for: Testing Associations of Plant Functional Diversity with Carbon and Nitrogen Storage along a Restoration Gradient of Sandy Grassland
Source: Front Plant Sci. 2016 Feb 19;7:189. doi: 10.3389/fpls.2016.00189 (PMC4759253; doi:10.3389/fpls.2016.00189)
Supplement: Supplementary file 5 [file Table_5.DOCX]

Table S5. Abbreviations of habitat, functional trait, functional diversity index, carbon storage and nitrogen storage.

| Variable | Abbreviation |
| --- | --- |
| ***Habitat*** |  |
| Mobile dune | MD |
| Semi-fixed dune | SFD |
| Fixed dune | FD |
| Grassland | G |
| ***Functional trait*** |  |
| Specific leaf area | SLA |
| Leaf dry matter content | LDMC |
| Leaf carbon content | LCC |
| Leaf nitrogen content | LNC |
| Root density | RD |
| ***Functional diversity index*** |  |
| Community-weighted mean | CWM |
| Single-trait functional divergence index | FDvar |
| Functional dispersion | FDis |
| ***Carbon storage*** |  |
| Aboveground standing biomass carbon | AGBC |
| Aboveground litter carbon | ALC |
| Belowground root carbon | BGRC |
| Belowground root carbon | STC |
| Belowground root carbon | TEC |
| ***Nitrogen storage*** |  |
| Aboveground standing biomass nitrogen | AGBN |
| Aboveground litter nitrogen | ALN |
| Belowground root nitrogen | BGRN |
| Belowground root nitrogen | STN |
| Total ecosystem nitrogen | TEN |
